# Can THEM6 targeting stop resistance to prostate cancer treatment?

**DOI:** 10.15252/emmm.202115504

**Published:** 2022-02-02

**Authors:** Mrittika Chattopadhyay, Doris Germain

**Affiliations:** ^1^ Tish Cancer Institute Division of Hematology/Oncology Icahn School of Medicine at Mount Sinai New York NY USA

**Keywords:** Cancer, Metabolism

## Abstract

Prostate cancer (PCa) clinical management relies heavily on androgen‐deprivation therapy (ADT). However, despite experiencing initial clinical benefit, patients getting ADT for non‐resectable PCa eventually relapse and develop fatal castration‐resistant PCa (CRPC). Multiple mechanisms of acquired resistance to treatment have been reported, including metabolic adaptation (Marine *et al*, 2020). Notably, activation of the endoplasmic reticulum (ER) unfolded protein response (UPR) has been associated with oncogenic transformation (Hart *et al*, 2012), tumor progression, metastasis dissemination, and resistance to therapy (Chen & Cubillos‐Ruiz, 2021). Targeting different branches of ER UPR has been found to be an effective tool against aggressive PCa (Nguyen *et al*, 2018; Sheng *et al*, 2019). Therefore, a better understanding of these pathways may lead to the identification of novel drug targets.

## Graphical abstract

D. Germain and M. Chattopadhyay discuss the study by Blomme *et al*, in this issue of EMBO Mol. Med., that shows that THEM6 reprograms lipid metabolism to support resistance to androgen‐deprivation therapy, and is a promising target for the treatment of castration‐resistant prostate cancer.

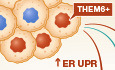

As the ER is the primary site for lipid and cholesterol biosynthesis, any change in lipid homeostasis can also induce ER stress and lead to subsequent activation of UPR. In this issue of EMBO Mol. Med., the study by Blomme *et al* (2022) compared different *in vivo* models of ADT and identified thioesterase superfamily member 6 (THEM6) as a potentially clinically relevant protein overexpressed in CRPC. THEM (thioesterase superfamily member) are type II acyl‐CoA thioesterases (ACOTs) which are engaged in deactivation of fatty acyl‐CoA thioesters, generation of free fatty acids and CoA, regulation of intracellular fatty acid (FA) trafficking, and are an integral part of ER lipid homeostasis (Tillander *et al*, 2017). However, the role of THEM6 in resistance to ADT had not been explored previously (Fig 1).

**Figure 1:**
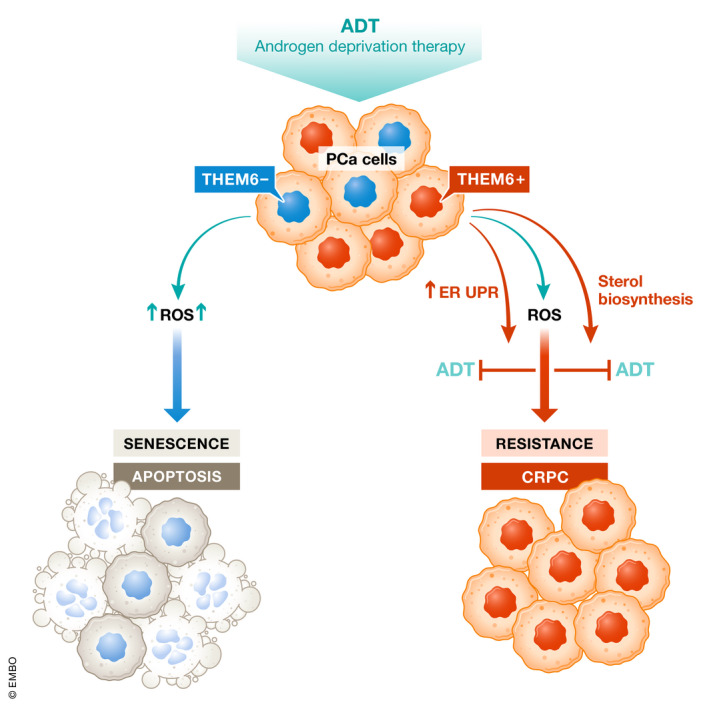
THEM‐6 in PCa cells is responsible for developing resistance to ADT by activating ER UPR and maintaining sterol biosynthesis.

In their study, Blomme *et al* (2022) show that THEM6 plays a critical role in ADT resistance by triggering ER stress response through preservation of ER membrane integrity, sterol biosynthesis, and ATF4 activation. To investigate the role of THEM6 in ADT resistance, the authors compared *in vivo* hormone naïve (HN) and castration‐resistant (CRPC) orthograft models of PCa by proteomics and found that THEM6 was significantly elevated in CRPC tumors. They further confirmed their observations *in vitro* in several PCa cell lines. To further explore the role of THEM6 in CRPC, the authors generated stable CRISPR‐based THEM6 knockout PCa cell lines (THEM6 KO). In a xenograft model, THEM6 KO significantly reduced tumor volume and increased tumor sensitivity to ADT. Previously, the authors had shown that ADT resistance was associated with rewiring of lipid metabolism (Blomme *et al*, 2020), and hence, they hypothesized that THEM6 was involved in the maintenance of tumor lipidome. Indeed, they found that loss of THEM6 in PCa cell lines resulted in a significant decrease in the intracellular levels of multiple triglyceride (TG) and ether lipid species and increased amounts of ceramides. They further confirmed *in vivo* by Raman spectroscopy that THEM6‐deficient tumors exhibited significantly less lipids and cholesterol. Further proteomics analysis of THEM6‐depleted prostate cancer cells revealed a significant downregulation of a large cluster of ER‐related membrane proteins. Electron microscopy analyses confirmed the morphological deformity of ER in THEM6‐deficient cells. As THEM6 is localized in the ER and plays an important role in maintaining lipid homeostasis, the authors hypothesized that it may also impact protein homeostasis. They performed pull‐down experiments in THEM6 overexpressing HEK293 cells, followed by MS analysis. Among the 152 proteins identified to interact with THEM6, several were involved in protein transport (exportins, importins, transportins, components of the ERAD machinery and the OST complex). The authors demonstrated that silencing THEM6 in PCa cell lines suppressed the expression of ER membrane‐associated chaperone calnexin without affecting its soluble homolog calrecticulin. These findings suggest that THEM6 is an ER protein and an important regulator of ER membrane integrity and trafficking. Since cholesterol acts as a precursor for *de novo* androgen synthesis and is required to sustain ADT resistance, a role of THEM6 in this process was investigated. The authors validated that loss of THEM6 reduced sterol and fatty acid synthesis in PCa cells. Using prostate adenocarcinoma (PRAD) TCGA dataset, the authors were able to establish a correlation between THEM6 and expression of several enzymes involved in the late steps of sterol biosynthesis. This analysis further revealed that fatty acid synthesis regulatory enzymes, acetyl‐CoA carboxylase and fatty acid synthase, were upregulated in THEM6‐enriched patient tumors. Collectively, these observations support the importance of THEM6 in regulation of ER protein and lipid homeostasis.

Disruptions of ER homeostasis in turn activate ER UPR to quickly relieve ER stress. THEM6 was essential for the initiation of ER UPR since THEM6‐deficient PCa cells were unable to activate ATF4/CHOP pathway. Further, THEM6‐deficient PCa cells were unable to activate UPR in response to palmitate‐induced ER stress, but initiated the UPR in response to hexadecylglycerol treatment, which is a precursor of ether lipid synthesis. This finding is of particular significance as it places THEM6 not only at the center of ether lipid metabolism but also as an important regulator of lipid‐mediated stress response. However, the exact molecular mechanism behind THEM6‐mediated activation of ATF4/CHOP pathway remains largely unclear.

The authors validated their findings in PCa patients and found a significant correlation between high THEM6 expression and shortened progression‐free and recurrence‐free survival in both the PRAD TCGA and the MSKCC (Taylor *et al*, 2010) cohorts. Further, UPR‐related genes were positively enriched in high THEM6 tumors, as well as in high THEM6 PCa patients.

In conclusion, the study by Blomme *et al* uncovered a novel role of THEM6 overexpression in ADT via its ability to promote persistent activation of ER UPR, which in turn facilitates PCa cells to survive therapy‐induced ER stress. Their findings establish THEM6 as a novel pharmacological target for cancer treatment.
